# Therapeutic effect on pyriform sinus carcinoma resection *via* paraglottic space approach

**DOI:** 10.3389/fsurg.2022.1068754

**Published:** 2023-01-06

**Authors:** Lei Wang, Dayu Liu, Ruijie Sun, Zhen Jiang, Jianlin Yue

**Affiliations:** Department of Otorhinolaryngology Head and Neck Surgery, Qilu Hospital (Qingdao), Cheeloo College of Medicine, Shandong University, Qingdao, China

**Keywords:** hypopharyngeal squamous cell carcinoma, pyriform sinus carcinoma, paraglottic space approach, laryngeal preservation, prognosis

## Abstract

**Objective:**

To analyse the surgical indications, surgical efficacy and key influencing factors of prognosis of using a novel surgical approach for pyriform sinus carcinoma resection utilising the paraglottic space.

**Methods:**

From 2014 to 2017, 93 patients with squamous cell carcinoma originating in the pyriform sinus were resected through the paraglottic space approach. The postoperative laryngeal function preservation, complications, survival rate and prognostic factors were analysed.

**Results:**

All patients were followed up for more than 5 years. The 2, 3 and 5 year overall survival rates of the patients were 77.2%, 61.6% and 47.4%, respectively. The univariate analysis of survival rate showed that primary tumour T stage and N stage had a statistically significant effect on the survival rate of patients (*P* = 0.047 and *P* < 0.001, respectively). Multivariate analysis with the Cox regression model revealed that N stage is an independent risk factor for postoperative survival (*P* = 0.042). The preservation rate of laryngeal function was 65.6% (61/93). Pharyngeal fistula incidence was 4.3% (4/93). Systemic distant metastasis and second primary cancer were found to be the main causes of death.

**Conclusions:**

As a novel surgical approach for the resection of pyriform sinus carcinoma, the paraglottic space approach can better expose the tumour, effectively improve the retention rate of laryngeal function, reduce the incidence of pharyngeal fistula and result in the better recovery of postoperative swallowing function with satisfactory long-term survival. N stage is an independent risk factor for postoperative survival.

## Introduction

Hypopharyngeal cancer is a highly malignant tumour of the head and neck that accounts for approximately 3%–5% of all head and neck malignancies (1). Its biological behaviour is poor, and its pathology is characterised by invasive growth and submucosal spread. It is prone to regional lymph node metastasis and invades other important structures in the neck. No specific symptoms can be observed in the early stage, and most cases are diagnosed in the middle and late stages because the location of the disease is hidden; therefore, the prognosis is poor (1–3). The pyriform sinus is the most frequently affected site and represents 70% of hypopharyngeal carcinoma (2). Given the close location of the pyriform sinus to the larynx, piriform sinus cancer easily invades the larynx, resulting in the destruction of laryngeal structure and function. Considering that accurate tumour resection is needed to achieve local control and improve survival rate, how to preserve laryngeal function as much as possible to improve the quality of life of patients after surgery is a difficult problem. Previously, we explored and summarised a new surgical approach, namely, the paraglottic space approach, through clinical practice to remove pyriform sinus carcinoma. This approach achieved good results in clinical practice. In this paper, we retrospectively analysed the clinical data of patients admitted to our department from 2014 to 2017 who underwent pyriform sinus carcinoma resection *via* the paraglottic space approach. The purpose of this study was to summarise and explore the surgical indications, therapeutic effects, surgical advantages and influencing factors of this approach for pyriform sinus carcinoma resection.

## Materials and methods

The clinical data of 93 patients who underwent primary pyriform sinus carcinoma resection *via* the paraglottic space approach in the Otolaryngology Head and Neck Surgery, from January 2014 to January 2017 were retrospectively analysed. All patients were operated for the first time without preoperative chemoradiotherapy and underwent flexible fiberoptic laryngoscopy, narrow band imaging, electronic gastroscopy and cervicothoracic computed tomography(CT) with contrast before surgery. Patients with distant metastasis or second primary cancer and general anaesthesia contraindications were excluded from this study. The age of the patients ranged from 41 years to 87 years. Amongst the 93 patients, 91 were males and 2 were females with an average age of 58.8 ± 9.51 years. The general clinical data and tumour characteristics of all patients (in accordance with the American Joint Committee on Cancer 2017 8th edition TNM staging of hypopharyngeal carcinoma) are summarised in Tables 1, 2.

**Table 1:** T N stage.

	N0	N1	N2	N3	
**T1**	5	2	0	0	7
**T2**	10	6	8	1	25
**T3**	7	9	25	2	43
**T4**	2	4	12	0	18
** **	24	21	45	3	

**Table 2:** Population and cancer data.

Data	*n* (%)	*X* ^2^	*P*
Gender		1.545	0.214
Male	91		
Female	2		
Age (year)		1.111	0.292
<60	50		
≥60	43		
Cigarette smoking		0.233	0.629
Ever	82		
Never	11		
Alcohol drinking		1.304	0.253
>50 g/d	84		
<50 g/d	9		
Tumour differentiation degree		3.909	0.142
Well	9		
Moderately	29		
Poorly	55		
Initial location of cancer in the pyriform sinus		2.612	0.271
Medial wall	56		
Lateral wall	18		
Lateral and medial walls	19		
Laryngeal function preservation	61	1.661	0.197
AJCC stage		7.017	0.071
** **I	5		
** **II	10		
** **III	24		
** **IV	54		

## Description of the surgical procedure

The neck dissection method was selected in accordance with the preoperative examination, primary tumour extent and regional lymph node metastasis. Patients with N0–N1 underwent ipsilateral II–IV selective neck dissection, patients with N2 underwent ipsilateral II–V neck dissection, and patients with N3 underwent ipsilateral I–V + contralateral II–IV neck dissections. If the tumour crosses the midline, bilateral neck dissection is performed; level VI and retropharyngeal lymph node dissections were performed for patients with T3–T4 and N2b or above.

## Tumour resection method

**Figure 1:**
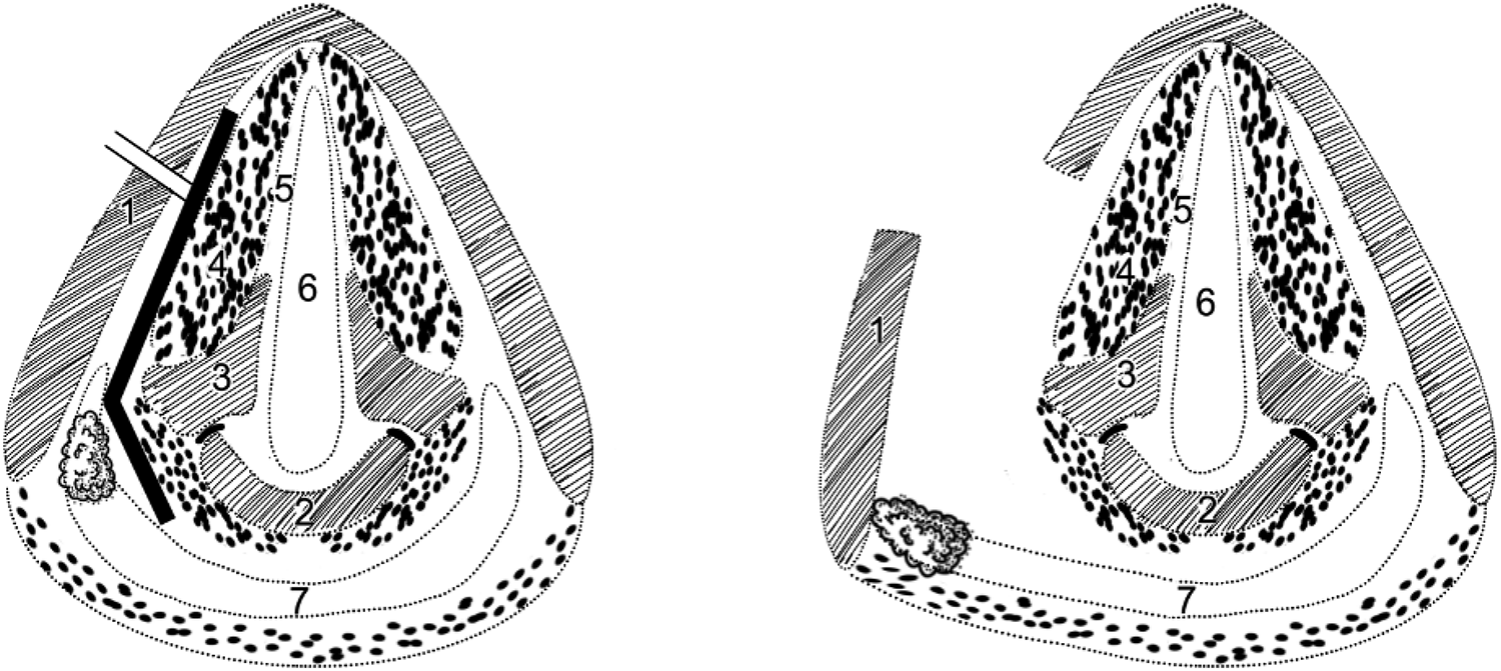
Schematic diagram of paraglottic space approach horizontal section of larynx at the level of the glotti 1 = thyroid cartilage, 2 = cricoid cartilage, 3 = arytenoid cartilage, 4 = thyroarytenoid muscle, 5 = vocal cord, 6 = airway lumen, 7 = lumen of the hypopharynx black solid line = paraglottic space, double slash = incision of thyroid cartilage.

After the neck lymph node dissection was completed, the free cervical sheath was pulled outward, the ipsilateral thyroid lobe was separated, and the ipsilateral great horn of the hyoid bone was removed. The inferior constrictor of the pharynx was cut off on the surface of the thyroid cartilage plate, and the thyroid cartilage plate was obliquely incised so that the thyroid cartilage plate was divided into two parts: anterior 2/3 and posterior 1/3. The piriform sinus was gradually separated from the larynx by pulling the posterior part of the thyroid cartilage plate outward and removing the connective tissue along the lateral surface of the thyroarytenoid muscle, and the paraglottic space was easily exposed (Figure 1).

For the carcinoma of the lateral wall of the piriform sinus, the thyroid cartilage plate was pulled outward, and the ipsilateral piriform sinus can be separated from the larynx through the paraglottic space approach. The tumour was fully exposed and resected under direct vision, and all the cutting edges were sent for frozen pathology until the negative margin was obtained. For the carcinoma of the medial wall of the piriform sinus, the lateral surface of the thyroarytenoid muscle was fully skeletonised until the arytenoid cartilage appeared. Firstly, the resection of the deep margin of the tumour was completed. Subsequently, the tumour was removed under direct vision by entering the pharyngeal cavity naturally through the free edge of the aryepiglottic fold or through the posterior incision of the thyroid cartilage plate into the pharyngeal cavity. If the paraglottic space was involved during the operation, partial laryngectomy or total laryngectomy was performed selectively.

## Repair

For stage T1 and T2 lesions, the pharyngeal cavity can mostly be closed with local mucosal pulling sutures after tumour resection. For stage T3 and T4 lesions, such as the tumour of the lateral wall of the piriform sinus invading the posterior wall of the hypopharynx or the tumour invading the entrance of the oesophagus, the defect of the lateral wall of the pharynx is so large that it is difficult to pull and suture directly after tumour resection. The most common method we use to repair the pharyngeal cavity is pedicled pectoralis major myocutaneous flap (Supplementary Video 1). In addition, after the pharyngeal cavity was closed, the ipsilateral uninvolved thyroid lobe was lifted and fixed on the lateral side of the pharyngeal suture to strengthen the pharyngeal wall and reduce the incidence of pharyngeal fistula.

## Postoperative adjuvant chemoradiotherapy

Postoperative radiotherapy alone was performed in 70 cases, whereas postoperative concurrent chemoradiotherapy was performed in 18 cases. The target areas of radiotherapy included the primary focus and the cervical lymphatic drainage area. All patients were treated with intensity-modulated radiotherapy (IMRT). The dose of radiotherapy was 50–66 Gy/25–33 f, 1.8–2.0 Gy/f, 5 f/W. The concurrent chemotherapy regimen was combined chemotherapy based on cisplatin.

Two patients refused radiotherapy, and three patients did not complete their radiotherapy programme. Two cases cited personal reasons. One case experienced pharyngeal fistula during radiotherapy, which improved after dressing change.

## Follow-up and statistical analysis

The survival stage and prognosis of all patients were followed up by means of telephone return visits, outpatient reexaminations, and other methods. Follow-up period was once every 3 months in the first and second years and once every 6 months in the third to fifth years. The re-examination included physical examination, laryngoscopy and imaging examination (cervicothoracic CT with contrast). Patients with clinically suspected tumour recurrence were examined by PET–CT. Survival rate was analysed *via* Kaplan–Meier analysis. The difference between groups was tested through log-rank method, and independent prognostic factors were analysed by Cox regression model in multivariate survival analysis. The statistical software SPSS 25 was used to analyse the data. *P* < 0. 05 was considered statistically significant.

## Results

### Postoperative data of patients

The follow-up period ended in December 2021, and all patients were followed up for 5 years. Six patients were lost by the end of follow-up. Survival analysis was performed in accordance with the final follow-up time. The follow-up time was 6–82 months, and the median follow-up time was 50 months. The patients who lost follow-up participated in the survival analysis as censored data, and the survival time was calculated according to the last follow-up time before the loss of follow-up. The postoperative pathology of all patients was squamous cell carcinoma. The metastasis rate of lateral cervical lymph nodes was 74.2% (69/93). Seventy patients underwent level VI lymph node dissection and retropharyngeal lymph node dissection. The positive rates of region VI lymph nodes and retropharyngeal lymph nodes were 12.9% (9/70) and 15.7% (11/70), respectively.

A total of 78 patients in this group had their larynges preserved, and all patients in T1 and T2 stages had their larynges preserved and underwent local mucosal flap repair of pharyngeal defects. Eleven patients with T3 and T4 stages underwent tongue root flap repair, 20 patients underwent pectoralis major myocutaneous flap repair, and 5 patients underwent subchin flap repair.

Pathological examination revealed that 81 patients (87%) had negative tumor margins without lesions. Twelve cases had positive or poorly defined margins microscopically, and in these cases, the margins of the frozen sections were expanded until the margins were negative.

Swallowing function. Modified barium swallow (MBS) and dysphagia score (DS) were used to evaluate swallowing function. Patients were advised to begin oral intake 2–3 weeks after surgery to promote laryngeal functional recovery. The patients began to eat large pieces of food by mouth, such as bread, and then gradually shifted to a half solid–half liquid diet. The result of the swallowing function assessment is shown in Tables 3, 4. Four patients with DS level 3 and two patients with DS level 4 underwent MBS assessment. The patients were given a liquid diet to assess whether food enters the airway and the severity of inhalation using the Penetration–Inspiration Scale (PAS). Amongst the four patients with DP level 3, one patient was under PAS1, one patient was under PAS2, and two patients were under PAS4. Amongst the patients with DP level 4, one patient was under PAS4, and one patient was under PAS5. Five patients who underwent laryngeal preservation had poor swallowing function. However, except for the patient with PAS5 grade, the gastric tube was removed before discharge. After 16–35 days (average 26 days) of operation, patients who could have the gastric tube removed.

**Table 3:** DS.

	Symptoms	T1	T2	T3	T4	
1	No symptoms.	7	22	28	3	
2	Rare cough during liquid food deglutition.		3	5	4	
3	Frequent cough during liquid food deglutition. Rare cough during solid food deglutition.			3	1	
4	Frequent cough during solid and liquid food deglutition.			1	1	
5	Frequent cough not related to food introduction. Inadequate food intake					
6	Recurrent aspiration pneumonia					

**Table 4:** The preservation of laryngeal functions in different T stage.

T grade	Laryngeal preservation		Laryngeal functions		
	Yes (78 cases)	No (15 cases)	Deglutition	Speech	Respiration
T1–T2	32	0	32	32	32
T3	37	6	35	37	26
T4	9	9	6	9	3
Total	78	15	73	78	61

Speech function. The speech function of postoperative patients was the same as that before operation. All patients were able to communicate with doctors and their families without barriers.

Tracheal cannula removal time. Amongst the 78 patients with laryngeal preservation, 63 patients were extubated after radiotherapy. Unfortunately, 2 patients underwent tracheotomy and intubation again with laryngeal edema. After 51–75 days (average 62 days) of operation, patients who could have the tracheal cannula removed.

According to the overall evaluation, 61 of the 78 cases with laryngeal preservation at the end of treatment retained laryngeal function (ability to speak, no tracheotomy, and deglutition without symptomatic aspiration, Table 4).

During the perioperative period, four cases had internal pharyngeal fistula, two cases experienced subcutaneous effusion, and one case experienced bleeding at the tracheotomy, all of which were cured by dressing change and pressure bandaging. One case of pulmonary infection improved after switching to sensitive antibiotics. No death occurred during the perioperative period.

### Survival and prognostic factor analyses

**Figure 2:**
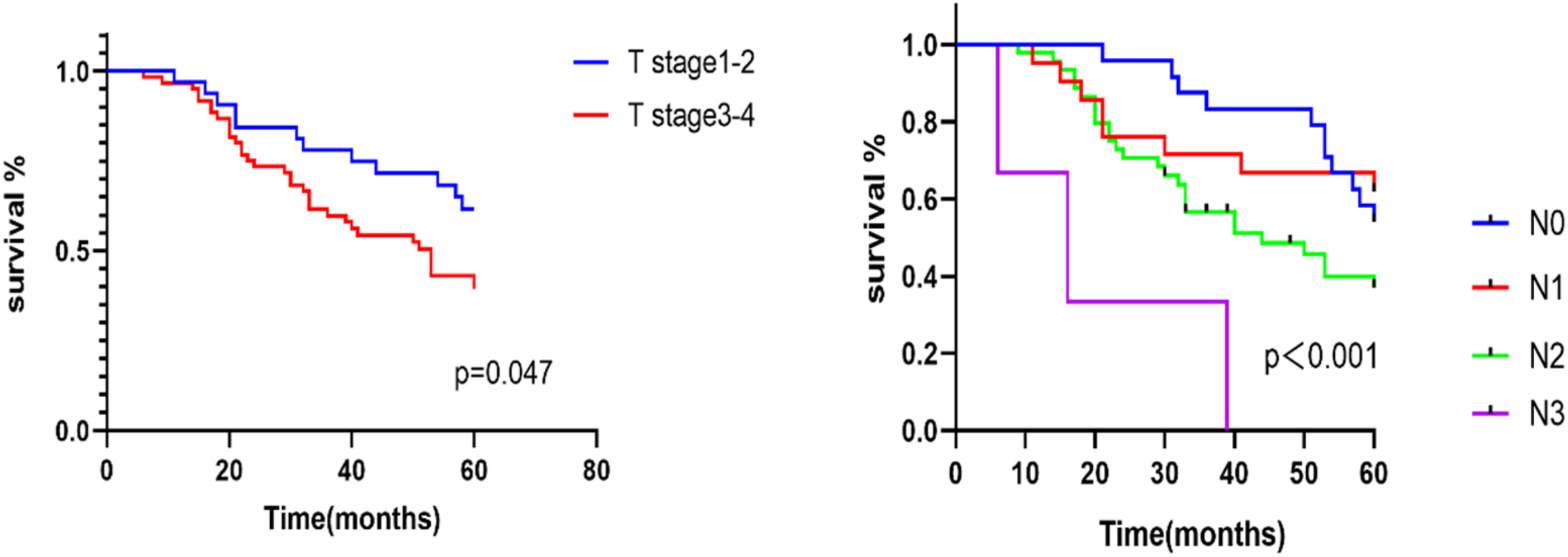
Kaplan-Meier survival curves for different T-stages and N-stages.

**Figure 3:**
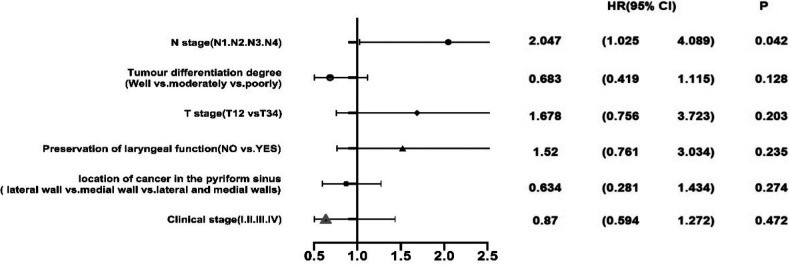
Multivariate regression analysis of factors influencing overall survival.

The Kaplan–Meier survival curve method provided a 2-year survival rate of 77.2%, a 3-year survival rate of 61.6% and a 5-year survival rate of 47.4%. Univariate analysis was carried out in accordance with the general data, tumour characteristics and TNM stage of the patients. The results of log-rank test showed that sex, age, history of smoking and alcohol, degree of tumour differentiation, primary site, clinical stage and preservation of laryngeal function were all *P* > 0.05, which indicates no statistical significance (Table 2). However, significant differences in survival were found between stages T1–T2 and T3–T4 (*χ*^2 ^= 3.959, *P* = 0.047) and amongst N0–N3 (*χ*^2 ^= 17.767, *P* < 0.001; Figure 2). The Cox regression analysis of tumour differentiation based on primary site, clinical stage, T and N stages and preservation of laryngeal function revealed that N stage is an independent risk factor of pyriform sinus carcinoma resected *via* the paraglottic space approach (*P* = 0.042, Figure 3).

### Cause of death

Fifty-one patients died as of the last follow-up date. The main causes of death included systemic distant metastasis (18 cases, 35.3%), including lung metastasis (9 cases), liver metastasis (5 cases), bone metastasis (3 cases) and brain metastasis (1 case); second primary cancer (11 cases, 21.6%), including middle and lower oesophageal cancers (7 cases), central lung cancer (3 cases) and gastric cancer (1 case) and nontumour causes of death (9 cases, 17.6%), including respiratory failure (3 cases) and cardiogenic death (2 cases), severe pneumonia (1 case), asphyxia caused by sputum thrombus (1 case) and unknown causes (2 cases). Local metastases of cervical lymph nodes were found in 7 cases (13.7%). Local tumour recurrence was found in 6 cases (11.8%).

### Case illustration

**Figure 4:**
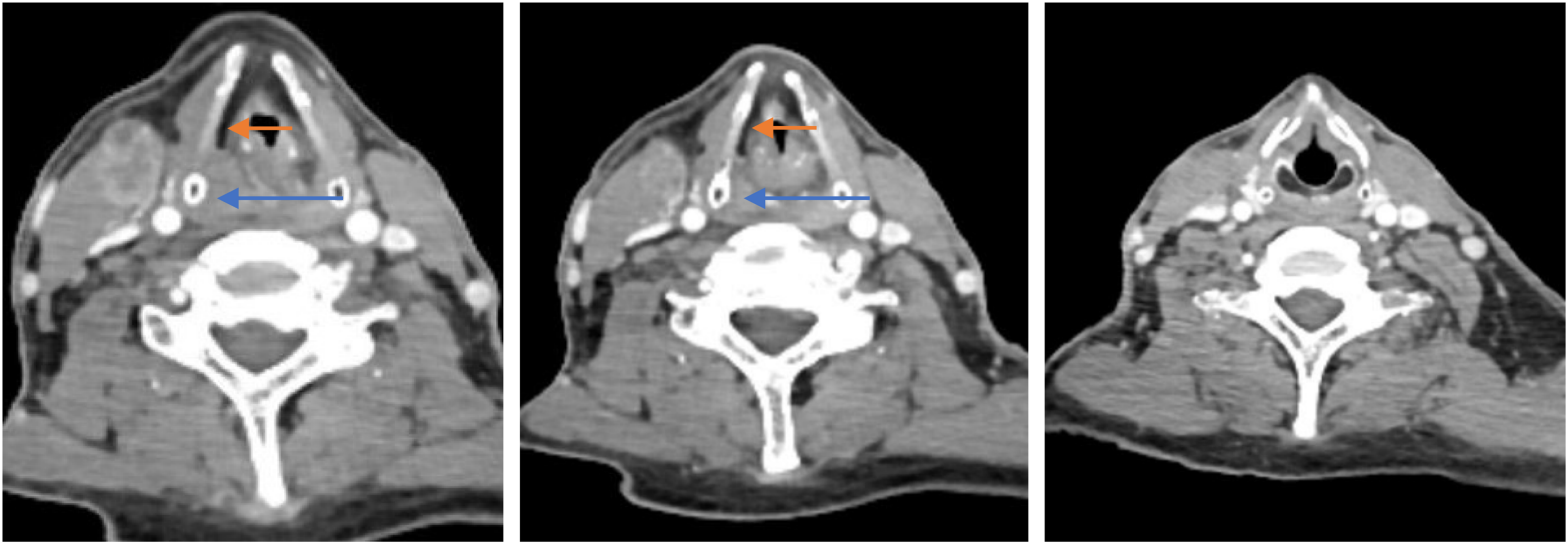
Preoperative contrast CT with contrast of one patient who was treated with the paraglottic approach. The paraglottic space was identified not completely invaded and the esophagus was not involved. The blue arrow indicates the tumor. The red arrow indicates the paraglottic space.

**Figure 5:**
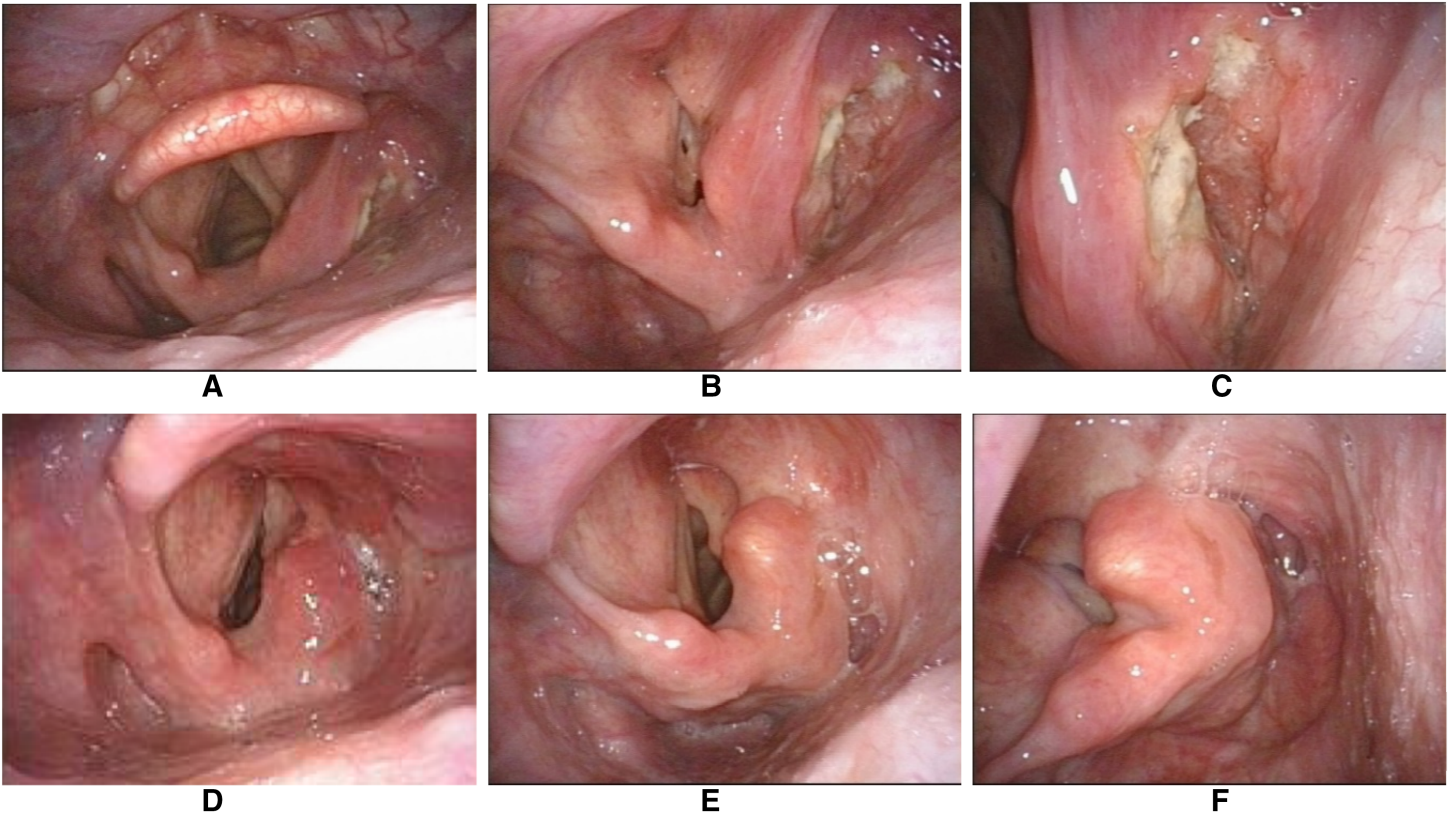
Representative pre-and postoperative laryngoscopic images of the same case. (**A–C**) The preoperative examination showed the tumor was confined to the right pyriform sinus without involving the laryngeal cavity. (**D**) The anastomotic stoma of the right pyriform sinus still appeared edematous 3 months after the operation. (**E,F**) No tumour recurrence at 2 years post-operative review.

**Figure 6:**
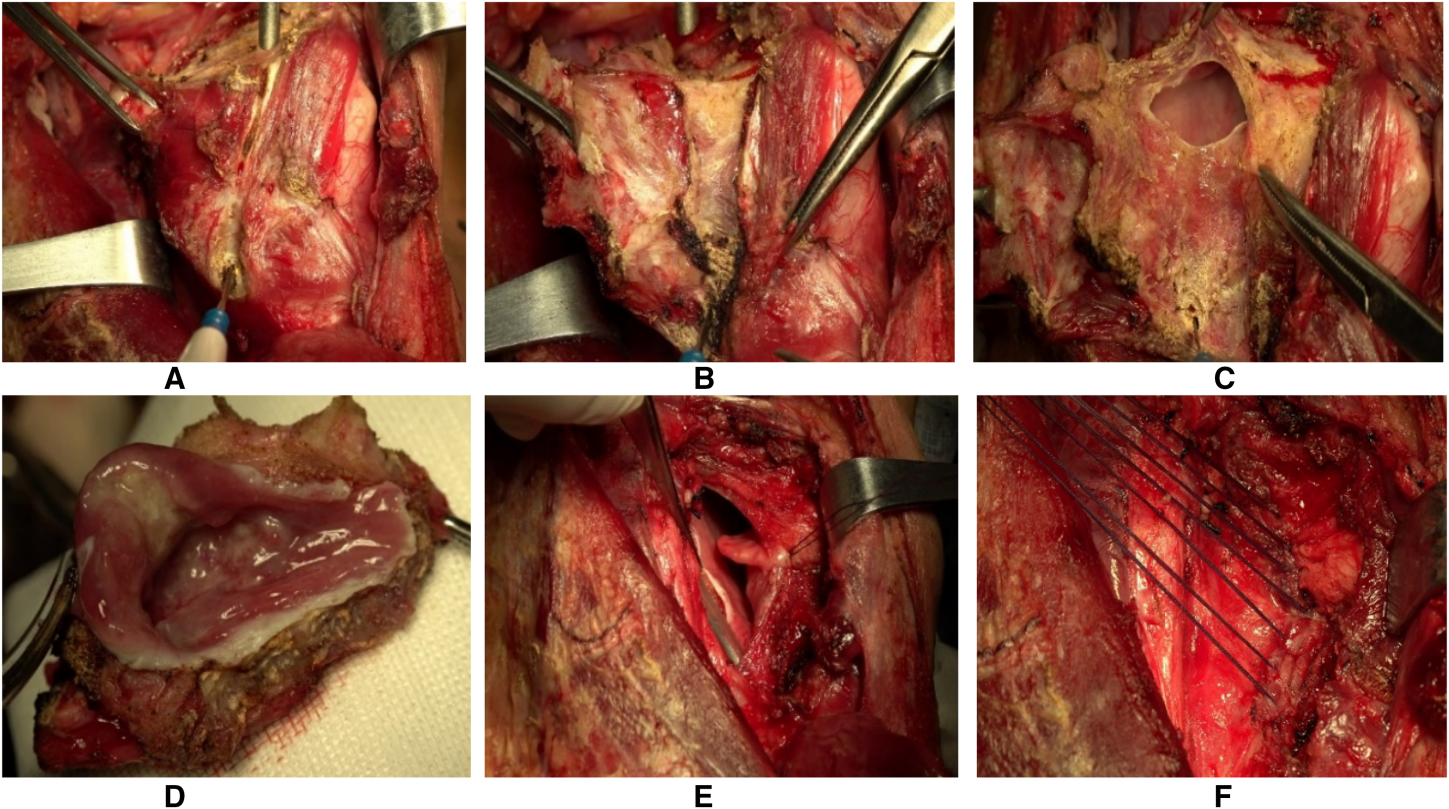
Pictures of surgical procedures. (**A**) Exposure of lateral edge of thyroid cartilage plate. (**B**) After the moderate outwards traction and lysis of the surrounding tissue, the parglottic space was exposed. (**C**) Enter the pharyngeal cavity from the paraglottic space and remove the tumour under direct vision. (**D**) Remove the tumour along the safe boundary of the tumour. (**E**) the mucosa of the posterior wall of the hypopharynx is sutured up and down to widen the pharyngeal cavity. (**F**) Suture the pharyngeal mucosa and close the pharyngeal cavity.

A 58-year-old male who underwent CT with contrast and flexible fiberoptic laryngoscopy (Figures 4, 5A–C) had a tumour that originated in the medial wall of the piriform sinus but did not invade the paraglottic space, larynx and oesophagus. The patient had a history of smoking for 30 years with an average of 15 cigarettes per day and a history drinking for 30 years with an average of 120 g/day. Tumour resection *via* paraglottic approach was performed to treat the tumour (Supplementary Video 2). The patient's postoperative diagnosis was hypopharyngeal squamous cell carcinoma (piriform sinus, T2N2bM0, IVa stage). Two weeks after operation, the patient was able to perform oral intake, and the gastric tube was removed. IMRT was performed 1 month after operation, then the tracheotomy tube was removed after radiotherapy, and the laryngeal function of the patients was preserved. Postoperative flexible fiberoptic laryngoscopy showed that the anastomosis was intact and no tumour recurrence occurred (Figures 5D–F). Critical surgical steps were shown in (Figure 6).

Notably, when the tumour was removed by utilising the paraglottic space, the normal pharyngeal mucosa was morepreserved, the suture tension ws reduced, and the new pharyngeal cavity had enough space. In addition, the thyroid lobe was lifted as a reinforcement plane for the new pharynx.

## Discussion

The paraglottic space is a potential space containing adipose tissue and blood vessels, bordered laterally immediately adjacent to the thyroid cartilage, medially adjacent to the thyroarytenoid muscle and bordered dorsally to the piriformis sinus (4). The paraglottic space can be easily exposed if the thyroid cartilage plate is cut obliquely during the operation and the posterior part of the thyroid cartilage plate is pulled outward (such as the description of the previous surgical method). The advantages of the paraglottic space approach are as follows. ①The paraglottic space is a natural anatomical space, and the tissue is easily separated through the paraglottic space approach with less intraoperative blood loss and a clear surgical field. ②The hypopharynx is easier to separate from the larynx and the highly wrinkled pharyngeal cavity is easily planarised to fully expose the tumour and removing the tumour in a 3D way under the full dissociation of the mucous membrane through the paraglottic space approach. The tumour is not squeezes in the process of surgical resection, which is in line with the principle of tumour resection. ③The surgical field of view *via* the paraglottic space approach is well exposed. Combined with the preoperative narrow band imaging examination, the tumour can be accurately removed under direct vision, and the normal hypopharyngeal mucosa can be preserved as much as possible, which is conducive to pharyngeal cavity reconstruction and function preservation.

The National Comprehensive Cancer Network guidelines point out that surgery is feasible for patients with T1 + N positive and T2−3Nx hypopharyngeal cancer to preserve the laryngeal structure, and partial laryngectomy (open or laser) combined with postoperative radiotherapy can be considered. These patients are expected to preserve laryngeal function.Relevant scholars also confirmed the feasibility of laryngeal function-preserving surgery for pyriform sinus carcinoma and provided a pathological basis (5–7). Although a part of pyriform fossa carcinoma belongs to T4, only the posterior edge of the thyroid cartilage, a small part of the cricoid cartilage, or the entrance of the cervical esophagus are involved. After tumor resection, most of the larynx can be preserved, the pectoralis major myocutaneous flap can be used to reconstruct the pharyngeal cavity, and the preservation of laryngeal function can be realised. We reviewed and compared literature on the surgical and non-operative treatments of advanced hypopharyngeal carcinoma in the past 5 years (Table 5) (8–12). No statistical differences were found in the overall survival rate between the two treatments, but the survival rate of surgical treatment was higher than that of non-operative treatment. Additionally, the preservation of laryngeal function has more advantages in non-operative treatment than in surgical treatment.

**Table 5:** Summary of literature on surgical and non-operative treatments of advanced hypopharyngeal carcinoma in the past 5 years.

Author	Stage	Treatment arms (*n*)	Overall survival	Follow up period
Reis et al (8)	III–IV	RT (81: ICT25\CRT73) vs. SRT (63)	47.5% vs. 55.6% (2 year) 29.2% vs. 32.8% (5 year)	3–110 months (Median 36.6 months)
Chung et al (9)	III–IV	ICT (74) + CRT (53) vs. SRT (139)	44.6%for ICT 39.6% for CRT 45.3% for SRT	6–156 months (Median 36.6 months)
Iwae et al (10)	III–IV T4a	CRT (127) vs. TPL (127) + po IC (23)RT (48) CRT (46) vs. TPL (46)	53.5% vs. 58.5% (5 year) 26.0% vs. 56.5% (5 year)	>1 year
Harris et al (11)	III–IV	CRT (48) vs. SRT (28)	41.3% vs. 66.3% (5 year)	6–120 months (Median 17 months)
Kim et al (12)	T2–3 T4a	CRT (522) vs. SRT (98) CRT (126) vs. SRT (111)	48.7% vs. 46.5% (3 year) 26.1% vs. 29.9% (3 year)	5 year

IC, induction chemotherapy; RT, radiation therapy; ICT, induction chemotherapy followed by (chemo)radiotherapy; CRT, chemoradiotherapy; TPL, total pharyngolaryngectomy; SRT, surgery-based therapy.

Literature review and NCCN guidelines suggest that surgical treatment combined with postoperative radiotherapy and chemotherapy is feasible for the treatment of patients with advanced hypopharyngeal carcinoma. A hypopharyngeal cancer art review (13) pointed out that future directions will continue to focus on surgical innovation to afford functional organ preservation while improving survival outcomes. We presented a new approach for HSCC resection *via* the paraglottic space for patients with surgical indications to provide a wider field of vision during operation, accurately remove hypopharyngeal carcinoma, protect the uninvaded laryngeal structure, and perform functional organ preservation. Compared with other literature, the preservation rate of laryngeal function is closest to that of related non-operative treatment (8–10), which provides a good idea for the surgical resection of hypopharyngeal carcinoma.

The paraglottic space of stage T1–T2 pyriform sinus carcinoma is rarely invaded. It is the best indication for this surgical approach. In addition, the paraglottic space approach can be used if the T3–T4 lesion does not involve the paraglottic space, because most cases of the vocal cord fixation is caused by tumour compression and not by tumour invasion of the paraglottic space (14). For the carcinoma of the lateral wall of the piriform sinus, the tumour rarely invades the larynx because of its anatomical position. The approach through the paraglottic space can fully separate the hypopharynx and larynx, that is, the entire tumour can be directly exposed from the ventral side. The surgical field of view is wide, and the tumour can be accurately removed. Especially if it is T1–2, the tumor can be easily approached into the pharyngeal cavity from the upper edge of the tumor without having to go through the paraglottic space, as invasion into the thyroid cartilage is rare.For the primary tumour in the medial wall of the pyriform sinus, the transglottic approach can firstly complete the resection of the deep cutting edge of the tumour when entering the pharyngeal cavity, then separate the tumour from the larynx by pulling the thyroid cartilage plate outward and remove the tumour under direct vision. Preoperative cervical CT with contrast and flexible fiberoptic laryngoscopy examination can effectively determine the presence or absence of paraglottic space and laryngeal cartilage involvement. If the paraglottic space is involved, in the actual surgical operation, we can first enter through the paraglottic space approach, separate the hypopharynx and larynx and then combine the lateral pharyngeal approach, and observe the scope of tumour invasion in multiple planes. Supraglottic hemilaryngopharygectomy, partial pharyngectomy or total laryngectomy is performed according to the actual extent of tumour invasion.

The Kaplan–Meier survival curve analysis of the patients who underwent paraglottic space approach for pyriform sinus carcinoma resection in this group found statistically significant differences between stages T1–T2 and T3–T4 and amongst different N stages. Cox multivariate analysis revealed that N stage is an independent risk factor. This result was consistent with the findings of related studies (15, 16). All these results indicated that N staging is of great importance to the prognosis of patients and suggested that the cervical lymph node recurrence of hypopharyngeal carcinoma should be controlled effectively in the clinic. Therefore, the preoperative CT with contrast of the neck and the neck ultrasound evaluation of N staging are particularly important. Special attention should be paid to the dissection of level VI and retropharyngeal lymph nodes during operation. Our experience is that level VI and retropharyngeal lymph nodes are dissected for patients above N2b. The main direction of invasion should be observed for T3–T4 tumours with a larger extent of invasion. Attention should be paid to the dissection of retropharyngeal lymph nodes for those invading the posterior wall of the pharynx, and attention should be paid to level VI lymph node dissection for those invading the entrance of the oesophagus downward. The affected side of level VI lymph nodes is thoroughly dissected strictly along the thyroid surgical capsule after dealing with the superior polar thyroid vessels of the thyroid gland of the affected side is not invaded. In addition, in this group of cases, patients with lymph node extracapsular spread, which was confirmed by postoperative pathology, were treated with simultaneous radiotherapy and chemotherapy after operation.

However, according to the preoperative evaluation, preserving laryngeal function should not be forced to the elderly who cannot eat normally and have systemic multiple organ diseases, especially those with severe pulmonary dysfunction, as it may cause serious complications, such as hypostatic pneumonia and even death (17).

Transoral robot pyriform sinus surgery (TORS) opens up a new surgical perspective and it is considered a kind of conservative procedure together with laser surgery. Systematic review and multicenter studies (18–20) shows that TORS oncology and functional indicators are ideal. However, most of the data come from early hypopharyngeal cancer cases with short follow-up time, lack of large sample long-term follow-up and comparative study with non-operative treatment in the same period. Moreover, the piriform sinus area is relatively narrow, and the anatomical position is deep; thus, good endoscopy and imaging are needed to evaluate the feasibility of the transoral exposure of tumors. The best surgical indications are small and superficial tumors (T1, T2) located in the upper part of the piriform sinus, and thyroid cartilage and T4 tumors are relatively taboo (20). Therefore, the limitation of TORS operation is relatively large. According to the research of Martin et al. and Weiss et al., the effect of trans-oral laser surgery seems to be less effective.With disease-free survival rate estimated, respectively, at 38% at 60 months and 37% at 45 months (21, 22).

In addition, the incidence of pharyngeal fistula in this study was 4.3%, which was considerably lower than those in other studies (23). As mentioned above, the paraglottic space approach can retain more normal hypopharyngeal mucosa, dissociate the pharynx from the larynx, effectively reduce the suture tension during pharyngeal reconstruction and prevent mucosal avulsion during pharyngeal movement. In addition, the lifting of the uninvaded lateral thyroid lobe to strengthen the pharyngeal wall is another main reason to reduce the occurrence of pharyngeal fistula. The lateral thyroid lobe has good blood circulation and large tissue volume, which can be used as the strengthening plane of the new pharyngeal cavity.

## Conclusion

In summary, the paraglottic space approach is a novel surgical approach, which can better expose the tumour. On the premise of ensuring a safe tumour resection margin, this approach has obvious advantages in preserving laryngeal function and reducing the serious postoperative complications of pyriform sinus carcinoma. It is beneficial in improving the quality of life of patients after surgery. This surgical approach can be applied in patients with lesions that do not involve the paraglottic space. N stage is an independent risk factor for postoperative survival. This study still has the deficiency of a relatively small sample size. Nonetheless, the resection method for primary postcricoid carcinoma and posterior pharyngeal wall carcinoma *via* the paraglottic space approach is worth further exploring and trying.

## Data Availability

The original contributions presented in the study are included in the article/Supplementary Material, further inquiries can be directed to the corresponding author/s.
